# Study of the burden on patients with chronic obstructive pulmonary disease

**DOI:** 10.1111/j.1742-1241.2008.01936.x

**Published:** 2009-01

**Authors:** J L Izquierdo, C Barcina, J Jiménez, M Muñoz, M Leal

**Affiliations:** 1 Sección de Neumología, Hospital General Universitario de GuadalajaraGuadalajara, Spain; 2 Departamento Médico, AstraZenecaMadrid, Spain

## Abstract

**Background:**

Health-related quality of life measures are widely used in patients with chronic obstructive pulmonary disease (COPD). However, they are extremely limited when used to evaluate patients outside the clinical trials. The aim of this study was to analyse the burden of the disease using a simple, validated, self-administered questionnaire specifically developed for patients in daily clinical practice.

**Methods:**

A total of 3935 patients (74.5% men; mean age, 67 years) participated in a cross-sectional study. The burden of COPD on patients was measured using the Clinical COPD Questionnaire (CCQ). COPD was rated at four levels by the forced expiratory volume in one second (FEV_1_) according to The Global Initiative for Chronic Obstructive Lung Disease (GOLD) scale.

**Results:**

The disease mainly affects old men (more than 50% were over 65 years of age) and non-employed men (23% were employed). Of the patients studied, 22.7% continued smoking, especially men (24.4% of men vs. 18.1% of women). Most patients (54%) were diagnosed with moderate stage II COPD. Severity of COPD was lower in women: 29.6% of men had severe COPD compared with 13.7% of women. During the last year, 65.1% had at least one acute exacerbation and 36.6% were admitted to hospital because of COPD exacerbation. No association was found between the body mass index and COPD stage. The variable that most influenced the disease burden was dyspnoea, as progression from grade 0 to grade 4 increased the disease burden by 1.78 points for symptoms, 2.43 for functional state and 1.53 for mental state. The functional classification of COPD also had a significant influence on the disease burden.

**Conclusions:**

The present findings show that dyspnoea and the degree of airflow limitation are the clinical variables that most affect the burden of COPD from the patient’s point of view.

**What’s known**

- From a clinical point of view, the impact of COPD in the patient depends on several variables.
- Quality of life questionnaires are complex and difficult to use in clinical practice.

**What’s new**

- Among several variables related with the clinical situation of a COPD patient, the present findings show that dyspnoea and the degree of airflow limitation are the clinical variables that most affect the burden of COPD from the patient’s point of view.
- A specific instrument called the Clinical COPD Questionnaire can be introduced in clinical practice as a simple tool to evaluate the global impact of the disease in each patient.

## Introduction

Chronic obstructive pulmonary disease (COPD) is one of the main causes of morbidity and mortality. It generates a considerable social burden and has an unfavourable outlook in terms of prevalence and mortality in the coming decades (1–5).

To date, several studies have analysed the financial, clinical and pharmacological aspects of COPD. Nevertheless, there is a little information on the most relevant aspects of the disease for patients, as the severity of the patient’s condition does not often correlate with the functional classification or the resources used. Therefore, in COPD, the term control usually has a different meaning for physicians, patients and the health authorities (6–9).

Although physicians and patients have not always agreed on this view of the condition, it has meant that in recent years, integrated management of COPD has begun to include patient-oriented variables. However, this approach has been limited by the lack of reliable validated tools that enable us to record the real needs of the patients.

In recent years, a solution has been developed in the form of questionnaires (generic and specific) examining the quality of life (QOL) and health status, and these have been used in patients suffering from respiratory conditions, especially asthma and COPD. However, these are complex, designed to evaluate populations and not subjects – with the result that they are of little use in clinical practice – and can underestimate the impact of the disease on the patient. In fact, COPD can affect lifestyle considerably, to the extent that patients often end up reducing their participation in many activities and restricting their social interaction (10–14).

To evaluate the burden of COPD on the patients, a specific instrument called the Clinical COPD Questionnaire (CCQ) has been developed and validated. It contains 10 items that are used to evaluate the general status of the COPD patient. The items are grouped in three domains: symptoms, functional state and mental state. The CCQ is self-administered and takes the patient no more than 2 min to complete (15,16).

This study aims to use this questionnaire to evaluate the burden of the disease in standard clinical practice and analyse its relationship with the clinical and functional aspects of severity, cultural factors and socioeconomic factors. This study represents the first use of the CCQ in clinical practice outside the context of a clinical trial, in our setting, and in Spanish.

## Materials and methods

Ours was a cross-sectional, epidemiological, observational clinical study carried out in Spain between January 2005 and February 2006 in pulmonology and primary care clinics among patients with a previous diagnosis of COPD. All patients were stable during the last month (clinical status and treatment had to be unaltered during the previous month) and were referred to outpatient clinics for regular monitoring. A total of 665 primary care doctors and 90 specialists contributed to the recruitment. Diagnosis and severity of the obstruction were established using the GOLD criteria. No spirometry was carried out during the study. To be included, patients had to have a previous diagnosis of spirometry-confirmed COPD. The values of this spirometry were used as reference values for establishing severity according to the GOLD classification. To avoid selection bias, the investigator included the first five patients fulfilling the selection criteria (Table 1). Additional cases were recruited by some doctors and they were also included in the study.

**Table 1:** Selection criteria

**Inclusion criteria**
All of the following inclusion criteria must be fulfilled:
Patients must be aged at least 40 years with diagnosed COPD in stages I–IV according to the GOLD guidelines
Patients must have been stable during the previous month, that is, with no change in their treatment for COPD
Patients must have given their informed consent to participate in the study
**Exclusion criteria**
Patients must not present any of the following criteria:
Terminal illness (life expectancy of < 6 months)
Diagnosed psychiatric condition or any other condition that modifies perceived health status or that prevents informed consent from being given
Inability to understand spoken or written Spanish
Modification of COPD treatment within the last month
Likely to be admitted to hospital or an institution at enrolment because of their COPD

COPD, chronic obstructive pulmonary disease; GOLD, The Global Initiative for Chronic Obstructive Lung Disease.

A single observation was made in which demographical data, data related to the study disease and socioeconomic data were collected. Patients completed the self-administered CCQ. Information was taken from the clinical history and the case report forms (CRF) designed for the study. The main investigator was in charge of collecting information from all the collaborating investigators, who were in turn responsible for including the patient in the study, obtaining informed consent and obtaining the necessary data, all in accordance with the study protocol. The study was approved by the Ethics Committee of Hospital de la Princesa (Madrid. Spain).

### Predetermination of the sample size

Disease burden was measured using the CCQ both globally and individually for each of the progressive stages of COPD (mild, moderate, severe and very severe). It was necessary to enrol 3800 patients to obtain a 95% confidence interval (CI) for the median score obtained from the CCQ. An estimated standard deviation of 1.7 was assumed, with an accuracy of 0.1 in the least frequent stage of COPD (very severe). The estimated level of losses was 10%. The ‘10% estimated losses’ refers to those on the CRF. This was because of the missing information that would have prevented us from having totally completed questionnaires.

### Measurement of disease burden using a specific instrument

The CCQ was originally validated for English-speaking countries and recently validated for Spain (submitted). It is composed of 10 questions using a Likert-type scale with scores ranging from 0 to 6 (never–almost always).

The replies are grouped into three domains – symptoms, functional state and mental state – all of which refer to the week before the questionnaire was completed. A global score is provided, and this is summarised as a measure of the disease burden ranging from 0 to 6. The CCQ is self-administered and takes the patient no longer than 2 min to complete. The questionnaire is self-applied and the investigators were given instructions to interfere as little as possible when the patient completed it. Dyspnoea was evaluated by the Modified Medical Research Council score.

The study was carried out according to the ethical principles established for research in human beings in the Declaration of Helsinki and its subsequent revisions. The protocol was approved by the Ethics Committee. Patients were included once written informed consent had been obtained.

### Statistical analysis

The primary variable (evaluation of the disease burden for a COPD patient measured by the CCQ) was analysed globally and individually for each of the progressive stages of COPD (mild, moderate, severe and very severe).

For the global score and that of each dimension, we calculated the mean, standard deviation, median, range and 95% CI. To identify the risk factors associated with the burden of COPD, we compared the mean scores of each dimension.

The *t*-test was used to evaluate significant differences for comparisons between two groups and a one-way analysis of variance for comparisons between more than two groups. We assumed a normal distribution because the global score and dimensions are a sum of items, and the analysed population size was large enough (Central Limit Theorem).

Multivariate models were constructed in which the burden of COPD was taken as a dependent variable, and the potential determinants (severity, socioeconomic factors and cultural factors) were included in the model as independent variables. Each of the variables was included in the model according to the results of the bivariate analyses. We deleted those variables in the model with the highest p-values, until all remaining variables had a p-value ≤ 0.20. The differences were only considered statistically significant if p < 0.05.

## Results

The study included 3935 patients with COPD of whom 316 were excluded for not fulfilling the eligibility criteria. When we had missing information because of incomplete questionnaires, the patient was also excluded. A total of 3619 patients (91.97%) enrolled by 755 physicians were analysed. Functional severity in both levels is showed in Table 2.

**Table 2:** Healthcare setting and COPD severity

	Stage I	Stage II	Stage III	Stage IV	Total
Primary care	649 (20.56%)	1755 (55.59%)	666 (21.09%)	87 (2.75%)	3157 (100%)
Pneumology	67 (16.22%)	173 (41.88%)	124 (30.02%)	49 (11.86%)	413 (100%)
Total	716 (20.05%)	1928 (54.00%)	790 (22.13%)	136 (3.80%)	3570 (100%)

Table 3 shows the demographical and clinical data. The disease mainly affected adult men (more than 50% were over 65 years old) who were not active (23% were working). Of the total number of patients, 22.7% continued smoking, especially men (24.4% vs. 18.1%). Most of the patients (54%) were diagnosed with moderate stage II COPD. Severity of COPD was lower in women: 29.6% of men had severe COPD compared with 13.7% of women. During the previous year, 65.1% experienced at least one acute exacerbation and 36.6% were admitted to hospital because of COPD. No association was found between body mass index (BMI) and COPD stage.

**Table 3:** Characteristics of the study population

	Total	Men	Women	p-value
No.	3619	2610	850	
Percentage		74.5	24.6	
Age [mean (SD)]	67.0 (10.8)	67.4 (10.4)	65.9 (11.9)	0.0002
BMI [mean (SD)]	27.9 (4.1)	28.0 (3.9)	27.8 (4.8)	0.2700
Smoking history (%)				< 0.0001
Non-smoker	20.5	7.3	63.2	
Ex-smoker	56.7	68.3	18.7	
Active smoker	22.7	24.4	18.1	
Education (%)				0.0002
None	20	18.5	25.2	
Primary	55.3	57.2	50.6	
Secondary	18.8	18.9	17.8	
Higher	5.9	5.4	6.4	
Activity (%)				< 0.0001
Employed	23	24.4	18.1	
Homemaker	11.7	0.5	46.9	
Unemployed	2.4	2.9	0.8	
Permanently disabled	7	8.5	2.6	
Retired	55.9	63.7	31.6	
COPD classification (GOLD criteria) (%)				< 0.0001
Mild COPD. Stage I	20.1	16.9	29.6	
Moderate COPD. Stage II	54	53.5	56.7	
Severe COPD. Stage III	22.1	25.2	11.8	
Very severe COPD. Stage IV	3.8	4.4	1.9	
Dyspnoea scale (%) (MMRC)				< 0.0001
Grade 0	19.9	17.9	25.9	
Grade 1	38.7	38.1	41.2	
Grade 2	23.5	25.0	18.3	
Grade 3	14	14.7	12.2	
Grade 4	3.9	4.3	2.4	
Number of acute exacerbations during the past year (%)				0.0050
0	34.9	38.3	34.8	
1	25	25.3	25.2	
2	18.8	19.1	18.7	
≥ 3	21.4	17.3	21.3	
Number of hospitalisations during the past year (%)				< 0.0001
0	63.4	69.1	63.4	
1	22.6	21.9	22.6	
2	8.7	5.4	8.7	
≥ 3	5.4	3.6	5.3	

BMI, body mass index; COPD, chronic obstructive pulmonary disease; MMRC, Modified Medical Research Council Score; GOLD, The Global Initiative for Chronic Obstructive Lung Disease.

As for pharmacological therapy, 79% of the patients analysed were taking inhaled corticosteroids, more than 60% were taking β_2_-agonists (short-acting or long-acting) and more than half (55.4%) were taking anticholinergic drugs.

**Figure 1:**
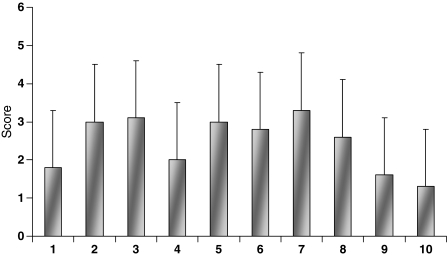
Mean score in the chronic obstructive pulmonary disease clinical questionnaire (mean ± SD) **On average, during the past week, how often did you feel:** 1. Short of breath at rest? 2. Short of breath doing physical activities? 3. Concerned about getting a cold or your breathing getting worse? 4. Depressed (down) because of your breathing problems? **In general, during the past week, how much of the time:** 5. Did you cough? 6. Did you produce phlegm? **On average, during the past week, how limited were you in these activities because of your breathing problems:** 7. Strenuous physical activities (such as climbing stairs, hurrying, doing sports)? 8. Moderate physical activities (such as walking, housework, carrying things)? 9. Daily activities at home (such as dressing, washing yourself)? 10. Social activities (such as talking, being with children, visiting friends/relatives)?

Figure 1 shows the average values for the CCQ in the whole population. The mean burden of the disease was 2.5. During the 7 days before the study, the patients analysed rarely felt depressed, sad, or that they could not breathe while resting because of their respiratory problems (mean response approximately 2). They were slightly limited (mean response approximately 2) in their daily or social activities during the 7 days before the study. For the remaining questions, their mean response varied around 3.

Women had a lower disease burden than men (2.32 ± 1.15 vs. 2.51 ± 1.22; p ≤0.0001). The disease burden of the younger patients was lower than that of the older patients (over 50 years of age). These differences were statistically significant. The Spearman correlation coefficient confirmed the association between age and disease burden. This association was positive in the sense that the higher the age, the greater the burden was. Non-smokers had a lower burden than ex-smokers: 2.37 ± 1.15 vs. 2.53 ± 1.23; p = 0.0066. It was observed that the patients who did not have a severe acute exacerbation or admission during the 12 months before the analysis presented a lower disease burden. These differences were statistically significant. The Spearman correlation coefficient confirmed the association between the number of admissions and the disease burden.

To study the factors affecting the disease burden, a multivariate linear regression analysis was carried out. This included those variables that had reached statistical significance in the bivariate analysis: sex, age, smoking, education, activity, years since diagnosis of COPD, COPD classification, dyspnoea scale, number of hospitalisations, number of acute exacerbations and treatment. The influence of all the variables on the disease burden was statistically significant, except for age, which was no longer statistically significant when it was evaluated along with the other variables. When we constructed the model with the variables that reached predefined statistical significance (p< 0.2), sex remained on multivariate analysis (although the difference between the sexes did not reach statistical significance). By contrast, age did not remain on multivariate analysis because it did not reach statistical significance (p= 0.62). The variable that most affected the disease burden was dyspnoea. When dyspnoea progressed from grade 0 to grade 4, disease burden increased by 1.98 points. In addition to this variable, functional classification of COPD was observed to affect the disease burden (Table 4).

**Table 4:** Disease burden. Total score

	Bivariate analysis				Multivariate analysis		
Source of variation	Mean	CI	P ANOVA or *t*-test	Adjusted mean*	CI	P compared with control group	General P**
Treatment			p < 0.0001				< 0.0001
Bronchodilator	2.6	(2.5–2.7)		2.79	(2.69–2.9)		
Bronchodilator + inhaled corticosteroids	2.8	(2.7–2.8)		2.84	(2.76–2.92)	0.4834	
Bronchodilator + oral corticosteroids	3.3	(3.2–3.4)		3.07	(2.97–3.17)	< 0.0001	
Sex			p < 0.0001				0.1667
Men	2.51	(2.46–2.55)		2.87			
Women	2.32	(2.24–2.40)		2.93	(2.84–3.03)	0.17	
COPD classification			p < 0.0001				< 0.0001
Mild COPD. Stage I	1.52	(1.45–1.60)		2.57	(2.46–2.68)		
Moderate COPD. Stage II	2.32	(2.27–2.36)		2.70	(2.62–2.79)	0.03	
Severe COPD. Stage III	3.41	(3.34–3.47)		3.05	(2.95–3.15)	< 0.0001	
Very severe COPD. Stage IV	4.02	(3.86–4.19)		3.28	(3.11–3.45)	< 0.0001	
Education			p < 0.0001				0.0004
None	2.81	(2.72–2.90)		3.04	(2.94–3.13)		
Primary	2.48	(2.42–2.53)		2.92	(2.84–3)	0.02	
Secondary	2.28	(2.19–2.37)		2.92	(2.82–3.02)	0.08	
Higher	1.91	(1.74–2.07)		2.72	(2.58–2.87)	0.00	
Activity			p < 0.0001				0.0934
Employed	1.83	(1.75–1.91)		2.81	(2.72–2.9)		
Homemaker	2.26	(2.14–2.37)		2.84	(2.72–2.96)	0.98	
Unemployed	2.54	(2.29–2.79)		3.02	(2.82–3.23)	0.15	
Permanently disabled	3.11	(2.97–3.26)		2.91	(2.78–3.04)	0.45	
Retired	2.72	(2.67–2.77)		2.92	(0–0.22)	0.07	
Dyspnoea scale (%) (MMRC)			p < 0.0001				< 0.0001
Grade 0	1.38	(1.31–1.45)		1.95	(1.84–2.07)		
Grade 1	2.14	(2.09–2.19)		2.39	(2.3–2.49)	< 0.0001	
Grade 2	2.91	(2.85–2.97)		2.89	(2.79–2.99)	< 0.0001	
Grade 3	3.58	(3.50–3.66)		3.34	(3.23–3.45)	< 0.0001	
Grade 4	4.44	(4.29–4.58)		3.93	(3.76–4.11)	< 0.0001	
Years since diagnosis			p < 0.0001				0.2021
0–10 years	2.24	(2.19–2.29)		2.88	(2.8–2.96)		
More than 10 years	2.79	(2.73–2.85)		2.92	(2.84–3.01)	0.20	
Hospitalisations			p < 0.0001				< 0.0001
None	2.05	(2.01–2.10)		2.80	(2.72–2.88)		
One or more	3.16	(3.10–3.22)		3.00	(2.92–3.09)	< 0.0001	
Acute exacerbations			p < 0.0001				< 0.0001
None	1.78	(1.72–1.84)		2.74	(2.65–2.83)		
One or more	2.82	(2.77–2.86)		3.06	(2.99–3.14)	< 0.0001	

* Minimum mean square. LS-mean. Adjusted for the remaining variables.

** For categorical variables, this is the p-value that contrasts if there are differences between all the categories of the variable. For ordinal variables, it is the p-value that contrasts if there is a linear association between the variable categories and the CCQ score. MMRC, Modified Medical Research Council score; COPD, chronic obstructive pulmonary disease.

The responses to the CCQ are grouped into three domains: symptoms, functional state and mental state. The variable with the greatest influence on disease burden in the three domains analysed individually was dyspnoea – when it progressed from grade 0 to grade 4, disease burden increased by 1.78 points for symptoms, 2.43 for functional state and 1.53 for mental state. Furthermore, functional classification of COPD also affected the disease burden (Tables 5, 6, 7).

**Table 5:** Disease burden – symptoms (multivariate analysis)

		Mean difference		
Source of variation	Adjusted mean*	Difference	95% CI	p-value
Treatment				< 0.0001
Bronchodilator	2.85			
Bronchodilator + inhaled corticosteroids	2.94	0.09	(−0.03–0.21)	
Bronchodilator + oral corticosteroids	3.17	0.32	(0.17–0.47)	
Smoking history				< 0.0001
Non-smoker	2.89			
Ex-smoker	2.94	0.04	(−0.08–0.17)	
Active smoker	3.13	0.24	(0.1–0.37)	
Sex				0.0294
Men	3.05			
Women	2.93	−0.12	(−0.22 to −0.01)	
COPD classification				< 0.0001
Mild COPD. Stage I	2.64			
Moderate COPD. Stage II	2.83	0.19	(0.05–0.33)	
Severe COPD. Stage III	3.18	0.55	(0.37–0.73)	
Very severe COPD. Stage IV	3.30	0.66	(0.37–0.94)	
Education				0.0727
None	3.07			
Primary	3.00	−0.07	(−0.18–0.04)	
Secondary	3.03	−0.04	(−0.18–0.1)	
Higher	2.84	−0.23	(−0.44 to −0.02)	
Dyspnoea scale (MMRC)				< 0.0001
Grade 0	2.13			
Grade 1	2.52	0.39	(0.24–0.53)	
Grade 2	2.98	0.85	(0.68–1.02)	
Grade 3	3.41	1.28	(1.08–1.48)	
Grade 4	3.91	1.78	(1.48–2.08)	
Hospitalisations				0.0033
None	2.92			
One or more	3.05	0.13	(0.04–0.22)	
Acute exacerbations				< 0.0001
None	2.80			
One or more	3.17	0.37	(0.28–0.45)	

* Minimum mean square. LS-mean. Adjusted for the remaining variables. MMRC, Modified Medical Research Council score; COPD, chronic obstructive pulmonary disease.

**Table 7:** Disease burden – mental state (multivariate analysis)

		Mean difference		
Source of variation	Adjusted mean*	Difference	95% CI	p-value
Treatment				< 0.0001
Bronchodilator	2.76			
Bronchodilator + inhaled corticosteroids	2.88	0.12	(−0.02–0.27)	
Bronchodilator + oral corticosteroids	3.18	0.42	(0.23–0.61)	
Smoking				0.0007
Non-smoker	3.07			
Ex-smoker	2.96	−0.11	(−0.28–0.05)	
Active smoker	2.78	−0.29	(−0.47 to 0.11)	
Sex				0.0002
Men	2.79			
Women	3.09	0.30	(0.14–0.45)	
COPD classification				0.0007
Mild COPD. Stage I	2.69			
Moderate COPD. Stage II	2.82	0.14	(−0.04–0.31)	
Severe COPD. Stage III	2.82	0.34	(0.11–0.57)	
Very severe COPD. Stage IV	3.22	0.53	(0.17–0.89)	
Education				0.0101
None	3.09			
Primary	3.01	−0.09	(−0.23–0.05)	
Secondary	2.93	−0.17	(−0.35–0.02)	
Higher	2.72	−0.37	(−0.64 to 0.1)	
Activity				0.0089
Employed	2.78			
Homemaker	2.79	0.01	(−0.24–0.27)	
Unemployed	3.07	0.30	(−0.09–0.69)	
Permanently disabled	3.10	0.32	(0.06–0.59)	
Retired	2.95	0.18	(0.01–0.35)	
Dyspnoea scale (MMRC)				< 0.0001
Grade 0	2.23			
Grade 1	2.50	0.26	(0.08–0.45)	
Grade 2	2.90	0.66	(0.45–0.88)	
Grade 3	3.29	1.06	(0.8–1.32)	
Grade 4	3.77	1.53	(1.15–1.91)	
Hospitalisations				< 0.0001
None	2.81			
One or more	3.07	0.26	(0.15–0.38)	
Acute exacerbations				< 0.0001
None	2.72			
One or more	3.16	0.44	(0.32–0.55)	

* Minimum mean square. LS-mean. Adjusted for the remaining variables. MMRC, Modified Medical Research Council score; COPD, chronic obstructive pulmonary disease.

**Table 6:** Disease burden – functional state (multivariate analysis)

		Mean difference		
Source of variation	Adjusted mean*	Difference	95% CI	p-value
Treatment				0.0005
Bronchodilator	2.70			
Bronchodilator + inhaled corticosteroids	2.71	0.01	(−0.1–0.12)	
Bronchodilator + oral corticosteroids	2.90	0.20	(0.05–0.35)	
Sex				0.0041
Men	2.71			
Women	2.83	0.12	(0.04–0.2)	
COPD classification				< 0.0001
Mild COPD. Stage I	2.38			
Moderate COPD. Stage II	2.50	0.12	(−0.02 to −0.02)	
Severe COPD. Stage III	2.92	0.54	(0.36–0.71)	
Very severe COPD. Stage IV	3.28	0.90		
Education				< 0.0001
None	2.95			
Primary	2.79	−0.16	(−0.27 to −0.05)	
Secondary	2.78	−0.16	(−0.3 to −0.03)	
Higher	2.56	−0.38	(−0.59 to −0.18)	
Dyspnoea scale (MMRC)				< 0.0001
Grade 0	1.59			
Grade 1	2.20	0.61	(0.47–0.75)	
Grade 2	2.77	1.18	(1.01–1.34)	
Grade 3	3.26	1.67	(1.48–1.87)	
Grade 4	4.03	2.43	(2.14–2.73)	
Years since diagnosis				0.1759
0–10	2.74			
More than 10	2.80	0.05	(−0.02–0.13)	
Age				0.0126
Under 70	2.72			
Over 70	2.82	0.10	(0.02–0.17)	
Hospitalisations				< 0.0001
None	2.63			
One or more	2.91	0.27	(0.18–0.36)	
Acute exacerbations				< 0.0001
None	2.67			
One or more	2.87	0.20	(0.11–0.29)	

* Minimum mean square. LS-mean. Adjusted for the remaining variables. MMRC, Modified Medical Research Council score; COPD, chronic obstructive pulmonary disease.

Although there were significant differences between the sexes, especially in the mental state domain (0.30; 0.14–0.45), these were not clinically relevant.

Patients treated in primary healthcare had a lower disease burden than those treated by specialist centres (2.42 ± 1.17 vs. 2.72 ± 1.40; p < 0.0001). These differences were maintained for symptoms (2.62 ± 1.24 vs. 2.95 ± 1.43; p < 0.0001), functional state (2.16 ± 1.30 vs. 2.53 ± 1.61; p < 0.0001) and mental state (2.54 ± 1.46 vs. 2.63 ± 1.40; p < 0.0001).

## Discussion

The most important contribution of this study is that, from the patient’s point of view, dyspnoea and degree of airflow obstruction are the clinical variables with the most relevance in disease burden. As the study is cross-sectional, this association does not necessarily imply causality. Other variables associated with the burden of COPD were sex, age, smoking, education, activity, years from diagnosis, number of acute exacerbations and number of hospitalisations. These findings are representative of a non-selected population attended by primary care physicians and pulmonologists in our setting.

The burden of COPD is considerable in all senses. In social terms, mortality is very high and will continue to increase in the coming years. Thus, it is currently the fifth cause of death in Spain and is expected to be the third cause of death in the world by the year 2020 (17,18).

Clinics are attending more and more patients suffering from this illness. In fact, visits to primary care centres because of COPD are two to four times more frequent than visits because of chest pain in patients with ischaemic heart disease (19). As far as health systems are concerned, the burden of COPD is increasing, mainly because of the costs generated by acute exacerbations and hospital treatment. In Spain, the annual cost of COPD is estimated to be €239 000 000, with hospitalisations making up 41% of the total cost (20).

From the patient’s point of view, COPD is responsible for the disability that restricts many daily activities. Today, QOL in COPD is an independent parameter of prognosis in the medium term (21). Nevertheless, despite the considerable burden of this disease, which affects all aspects of life, it seems that its impact is not taken as seriously as it should be. COPD symptoms are usually attributed to the effect of smoking, without it being appreciated that a well-defined, specific medical condition has developed. This ignorance of what COPD is on the part of the patient leads to a delay in visiting the doctor and receiving adequate treatment to improve symptoms and health-related QOL. When COPD is eventually diagnosed, the disease may already be very advanced with severe involvement of lung function.

The CCQ has been developed to measure the clinical control of COPD by covering the primary aspects of the disease (symptoms) as well as the secondary variables directly related to the process and which are very relevant from the clinical point of view (mental aspects). This tool enables us to assess how COPD is monitored; therefore, it is very useful in the clinical evaluation of the patient and in the analysis of the response to different therapeutic measures. In fact, the CCQ has proven to be very sensitive for detecting clinical improvements after smoking cessation (16).

Unlike generic or specific QOL questionnaires, the CCQ is designed to evaluate specific patients, but not populations, and it does not aim to evaluate the degree of well-being of the patients, who may frequently be affected by aspects not connected with the condition itself (16). In practice, COPD has a considerable impact on lifestyle, as it reduces the patient’s ability to participate in all types of activity and restricts social interaction.

This study reports the first use of the CCQ in the clinical practice outside the context of a clinical trial; therefore, it enables us to measure the burden of the disease in our setting from the patient’s perspective and to discover which variables are clinically relevant in this burden.

Dyspnoea is the variable with the most impact on disease burden. This is the main symptom of COPD patients; it limits activities that require physical exertion and, when it is intense, can lead to disability (22). In practice, the progression from grade 0 dyspnoea to grade 4 dyspnoea means that the burden of the disease increases globally by 1.78 points, especially with regard to symptoms. Each increase in the score represented an increase in the burden of more than 0.40, which is the limit considered as clinically significant (16).

The relationship between dyspnoea and the severity of obstruction is weak. This is because of the complexity of the factors that intervene in the onset of dyspnoea, which can be physiological and psychological (22,23). FEV_1_ is an excellent parameter for evaluating the degree of functional involvement in COPD patients. It is also used to evaluate the efficacy of bronchodilators and can predict the mortality of COPD. However, unlike reports on quality-of-life questionnaires, which may be affected by factors that have nothing to do with the disease or the patient, our study showed that FEV_1_ expressed as a percentage of the theoretical value plays a more important role in COPD burden than has been appreciated in recent years. In clinical practice, changes in disease burden are particularly relevant when the patient progresses to stages III and IV.

When exacerbations appear, the normal life of these patients is even more altered and those with severe alterations can even fear for their life. Severe exacerbations affect health over a long period, even after they have been satisfactorily managed. Visits to the emergency department because of an acute exacerbation and subsequent admissions to hospital generate greater prescription of drugs for these patients (24). However, the episodic nature of an acute exacerbation could mean that, from the patient’s perspective, its impact is lower than that of dyspnoea and functional deterioration, which manifest themselves on a daily basis.

Malnutrition has been associated with a reduction in the different respiratory function parameters and with a deterioration in dyspnoea and QOL in COPD patients, and is an element of the BODE index (25–27). In our study, the percentage of underweight patients was low, similar to that detected in other studies carried out in Spain, although it is clearly lower than that found in other countries (28,29). We found no association between the BMI and disease burden.

In a recent study (24), sex was a predictor of drug consumption in COPD patients, and this could indicate differences in burden. Although there were significant differences between the sexes, especially in the mental state domain, these were not clinically relevant.

The number of non-smoker women in this study was very high. We cannot exclude that there may have been an error of interpretation of the question if she was an ex-smoker. However, In Spain, COPD in women (defined as cigarette smoke related disease) is still low but there are many old women (non-smokers) with chronic airflow limitation. We have no further details but, like in another countries, other diseases such as wood smoke exposure, previous pulmonary tuberculosis or long life asthma, can be the potential causes of chronic obstructive disease in our country and can explain, at least in part, the result (30,31). Regarding this topic, Birring et al. (32) observed that COPD in non-smokers predominantly affects women and has at least two pathological subgroups, one of which may be associated with organ-specific autoimmune disease. As the authors pointed out, further investigation of this group may disclose novel mechanisms of fixed airflow obstruction, but so far they are considered as COPD.

In conclusion, in our setting, dyspnoea and the degree of airflow obstruction are the clinical variables most associated with disease burden from the patient’s point of view. Unlike specific QOL questionnaires, in clinical practice, CCQ enables us to evaluate patients quickly and gives the advantage of informing us about the patient’s perception of the disease. In fact, COPD has an important effect on patients’ QOL, to the extent that participation in many types of activity and social interaction are reduced. This limits the clinical usefulness of QOL questionnaires.
